# A Photonic crystal fiber with large effective refractive index separation and low dispersion

**DOI:** 10.1371/journal.pone.0232982

**Published:** 2020-05-14

**Authors:** Zhihao Geng, Ning Wang, Keyao Li, Hui Kang, Xun Xu, Xing Liu, Weicheng Wang, Hongzhi Jia

**Affiliations:** Engineering Research Center of Optical Instrument and System, Ministry of Education, Shanghai Key Lab of Modern Optical System, University of Shanghai for Science and Technology, Shanghai, People’s Republic of China; Universidad Miguel Hernandez de Elche, SPAIN

## Abstract

A photonic crystal fiber (PCF) structure with a ring-core and 5 well-ordered semiellipse air-holes has been creatively proposed. Through a comparison between the structures with a high refractive index (RI) ring-core and the structure without, it conclude that a PCF with a high RI ring-core can work better. Schott SF57 was elected as the substrate material of ring-core. This paper compares the effects of long-axis and short-axis changes on the PCF and selects the optimal solution. Especially TE_0,1_ mode’s dispersion is maintained between 0 and 3 ps / (nm · km) ranging from 1.45 μm to 1.65 μm. This property can be used to generate a supercontinuum with 200 μm long zero dispersion wavelength (ZDM). In addition, Δn_eff_ reaches up to 10^−3^, which enables the near -degeneracy of the eigenmodes to be almost neglected. The proposed PCF structure will have great application value in the field of optical communications.

## 1. Introduction

Orbital angular momentum (OAM) beams have attracted great attention [1–9] since Allen et al. experimentally verified their existence in 1992 [1]. In the paraxial approximation, an OAM beam is characterized by a helical phase front of exp(jlφ), where l represents the topological charge value, and φ represents the azimuthal angle [7, 10]. As one of the most fundamental physical quantities in classical and quantum electrodynamics, OAM beams have been applied in many fields, such as optical communications [4], quantum information processing [5], and optical manipulation [6, 7]. These beams also have some unique characteristics. For example, compared with spin angular momentum (SAM) beams, OAM beams can accumulate and rotate exponentially, which has prompted promising research and applications in quantum entanglement [11, 12], optical imaging [13], high capacity optical communication [14–16] and other fields. Moreover, with the growth of Internet users, the requirements for transmission capacity and channel segmentation have become higher and higher, and various tests have been conducted on optical fiber communication technologies. To address the insufficiency of the transmission capacity, many effective technical measures in different dimensions have been proposed, such as mode division multiplexing [15–17], space division multiplexing [18], time division multiplexing [19] and wavelength division multiplexing [20]. Mode multiplexing of OAM, a new technology development in recent years, theoretically makes it possible to transmit an infinite number of OAM modes in a photonic crystal fiber (PCF). Therefore, it is necessary to study the OAM mode transmission in PCF fiber.

PCFs are regarded as a promising fiber technology that has some unique features, such as controllable nonlinearity, dispersion [21, 22], and an endless single mode [21]. Compared with traditional fibers, the structure of PCF can be designed more flexibly. The parameters of the mode can be optimized by adjusting the arrangement and size of the air holes. In addition, the number of OAM modes can also be changed with the air holes. As a result, there is a demand for a PCF that can steadily transport OAM modes as much as possible. In other words, a PCF must have a transmission capability that ensures a low confinement loss (CL), large mode area, low dispersion and low mode crosstalk for the OAM mode.

In 2013, P. Gregg et al. proposed a PCF that can support stable transmission of 12 OAM modes [23]. The transmission capacity of this PCF is severely limited by the insufficient number of OAM modes. Increasing the number of OAM modes for PCF fiber transmission has become a focus of attention. W. Tian et al designed a C-PCF which can support 26 OAM modes in 2016 [24]. X. Xu proposed a circular PCF based on silicon to transmit 30 OAM modes [7]. In addition, Z. A. Hu et al. designed a PCF supporting 26 OAM modes [26]. These authors chose As_2_S_3_ as a substrate material due to its high refractive index (RI); the large effective refractive index separation (Δn_eff_) between the HE_m+1, n_ mode and EH_m+1,n_ mode can effectively reduce the degeneracy between eigenmodes. Simultaneously, the intrinsic attributes of As_2_S_3_, namely its high material dispersion and large nonlinear RI, determine that it is not conducive to mode transmission.

**Fig 1:**
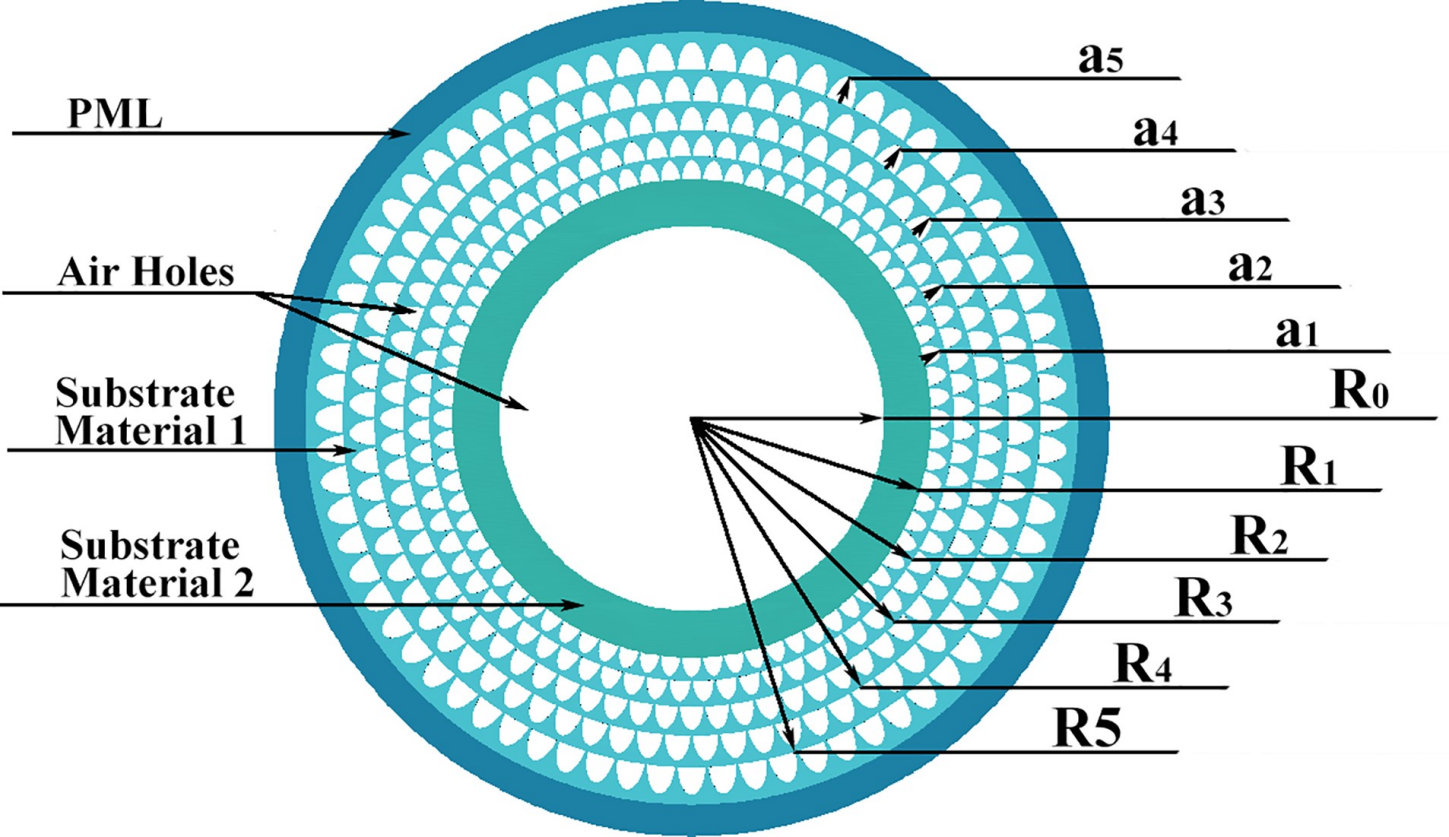
Schematic diagram of the designed PCF.

In this paper, we have further optimized based on previous literature [25]. We described a semielliptical PCF structure with a high RI ring-core between a central air hole and cladding in more detail. More remarkably, we also used a method for which the substrate material of the ring-core is different from that of the cladding [25]. First, we modified the structure in Reference [26] so that the materials of ring core and cladding are different. The material of ring core has a higher refractive index than the material cladding. We compared the performance between the structure with a high RI ring-core and the structure (in reference [26]) without a high RI ring-core, which can verify the improvement for this method. Then, we used a material in ring core with a high refractive index in the PCF structure shown in Fig 1 and further analyzed the performance of the proposed structure in detail.

## 2. Structural parameters, materials and methods

Fig 1 shows a PCF structure with semielliptical pores. This structure consists of a central pore, five semi-elliptical pores, and an outermost perfectly matched layer (PML). R0 shows the radius of the central air hole, and R_1_ to R_5_ represent the radii of the five air hole layers. Next, a_1_ to a_5_ are the semimajor axes of the air holes. In addition, the minor axis of each ellipse is half of the major axis. Table 1 shows the parameters for the PCF structure. Fused silica (n = 1.45 at a wavelength of 1.55 μm) was selected as the substrate material 1. A commercially available lead-silicate glass, Schott SF57, was chosen as the substrate material 2 in the ring-core due to its high linear RI (n = 1.8 at a wavelength of 1.55 μm) and low nonlinear RI (n_2_ = 10^−19^ m^2^w^-1^) [27]. The proposed PCF structure was numerically simulated by using the finite element method (FEM) and PML with COMSOL Multiphysics 5.2. The FEM, which greatly improves the calculation precision and reduces the computation time, is advocated by COMSOL. The PML is used as an absorbing boundary condition, and it is efficacious in preventing distortion of the electromagnetic field in the inner space of the PCF.

**Table 1:** Structure parameters for the proposed PCF.

Parameters	Refractive index	a_1_	a_2_	a_3_	a_4_	a_5_
Values (μm)	1.8	0.6125	0.665	0.735	0.805	0.875
Parameters	R_0_	R_1_	R_2_	R_3_	R_4_	R_5_
Values (μm)	6.7	8.3	9.1	10	11	12.1

## 3. Numerical results

By coherently combining the EH modes and HE modes with the same topological charge number l, the OAM modes are constituted by the following formulae [28]:

$$OAM_{\pm \text{l},m}^{\pm }=HE_{l+1,m}^{even}\pm jHE_{l+1,m}^{odd}$$ (1)

$$OAM_{\pm l,m}^{\mp }=EH_{l-1,m}^{even}\pm jEH_{l-1,m}^{odd}$$

where the superscript in the OAM modes is the direction of the circular polarization, and subscript ±l is the topological charges, which indicates the direction of the wavefont rotation. The direction of the wavefront phase rotation changes with circular polarization.

### 3.1. Comparison between the structure with a high RI ring-core and the structure without a high RI ring-core

**Fig 2:**
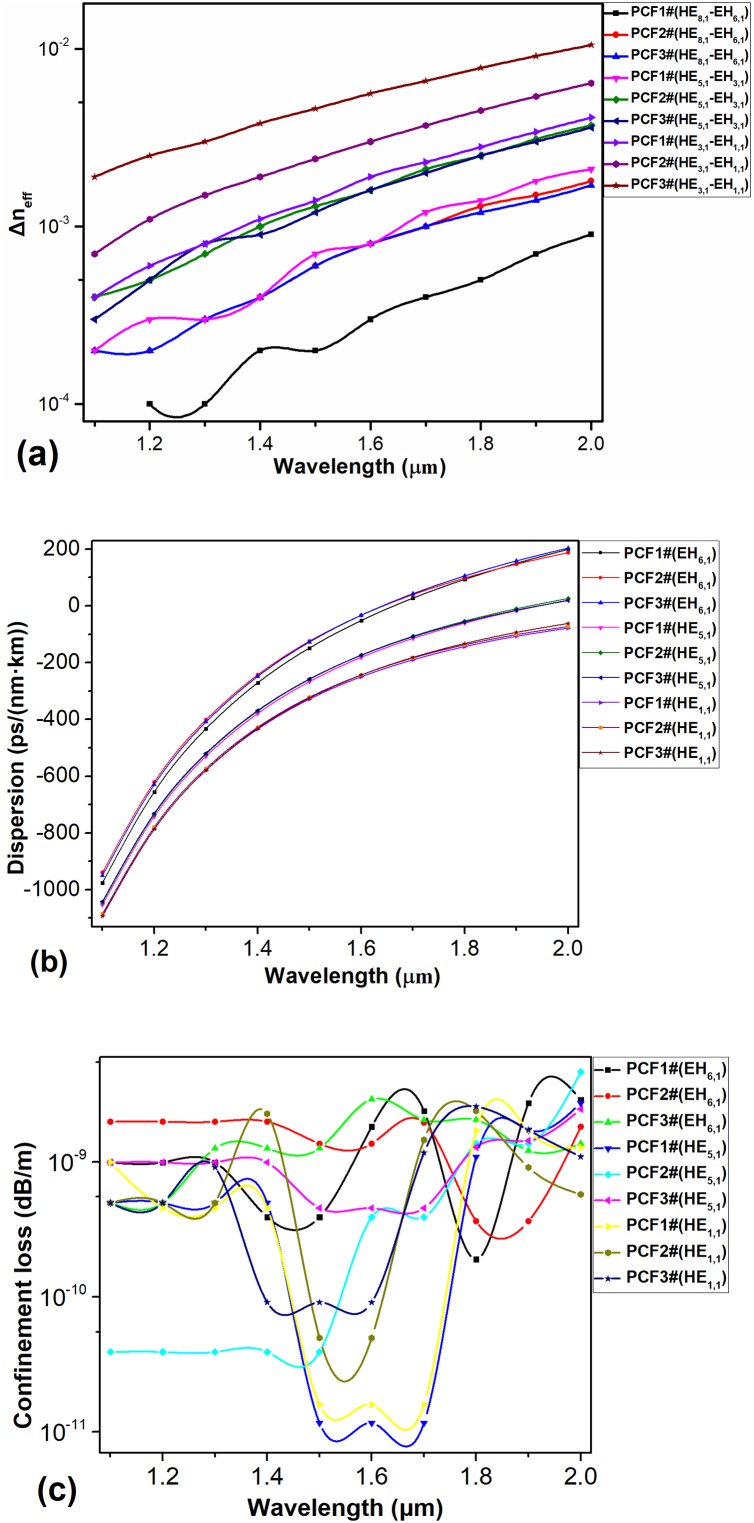
(a) Δn_eff_ of the partial eigenmodes in PCF#1, PCF#2, PCF#3; (b) Dispersion of partial eigenmodes in PCF#1, PCF#2, PCF#3. (c) Confinement Loss of partial eigenmodes in PCF#1, PCF#2, PCF#3.

We used a method of adopting a higher RI substrate material in the ring-core compared with the cladding [25] and further discussed its impact in many ways, which have not been mentioned previously. To verify the feasibility of this method, the PCF structure in reference [26] was selected as a sample. Significantly, when the RI of the substrate material in the cladding is greater than that of the ring-core, light beams will transmit in the cladding, resulting in a great confinement loss, especially for long-distance transmission. Thus, we should select a PCF whose RI of the substrate material in the cladding is less than that of the ring-core. For more persuasive results, we carried out two groups of analyses. As the substrate material used for the PCF structure in reference [26] is As_2_S_3_, a kind of chalcogenides glass with high RI, we chose a substrate material for the cladding with a relatively low RI. The substrate material of the cladding in the first group is fused silica, while that in the second group is Schott BAFN6 [29]. In addition, As_2_S_3_ is used as the substrate material for the ring-core in the two groups. In other words, the structure and material of PCF#1 are completely consistent with the literature [26]. The structure and material of the ring core of PCF#2 and PCF#3 are the same as those in literature [26]. The materials in the cladding are based on fused silica and Schott BAFN6, respectively. The comparison results are shown in Fig 2(a) and 2(b).

The Δn_eff_ between HE_m+1, 1_ and EH_m-1, 1_ for the eigenmodes is shown in Fig 2(a). In the case of the same eigenmode, all the Δn_eff_ for the eigenmodes in PCF#2 and PCF#3 are greater than that in PCF#1.

The Δn_eff_ between HE_3, 1_ and EH_1, 1_ in PCF#2 and PCF#3 displayed in Fig 2(a) have a value of almost above 10^−3^, with a maximum of up to 10^−2^, which is a considerably higher value than that in PCF#1. In addition, PCF#2 and PCF#3 possess dispersion and confinement loss curves similar to that of PCF#1, as demonstrated in Fig 2(b) and 2(c). The number of supported OAM modes for PCF#2 and PCF#3 is 30, which is improved compared with that of PCF#1. Through the comparative analysis above, it can be concluded that adopting a ring-core material with a high linear RI, low nonlinear RI and material dispersion can effectively improve the performance of the PCF. Based on this conclusion, we continue to explore ways of enhancing the performance of the PCF.

### 3.2. Structural optimization

After demonstrating that the differentiated design of the materials of ring core and cladding is beneficial to supporting OAM modes, we applied this method to the PCF structure shown in Fig 1. The material of the ring core is Schott SF57, and the material of cladding is silica. Based on this, we will further discuss this PCF structure. In section 3.2 and later parts, the main structure discussed is the PCF structure shown in Fig 1.

To further prove the optimality of the structure, this article changes the K value of the cladding air holes of the structure (K is the ratio of the major axis to the minor axis for the cladding air holes). The lengths of the long axes and short axes for each layer of air holes are shown in Table 2:

**Table 2:** The parameters for the air holes in each layer.

Number of layers	1	2	3	4	5
Long axis (μm)	0.6125	0.665	0.735	0.805	0.875
Short axis (μm)	0.35	0.38	0.42	0.46	0.5

Based on these parameters, the length of the long axis of each layer of air holes is adjusted, and the short axis is invariant (initial K value is 1.75). The K value varies from 1 to 2 in steps of 0.25.

**Fig 3:**
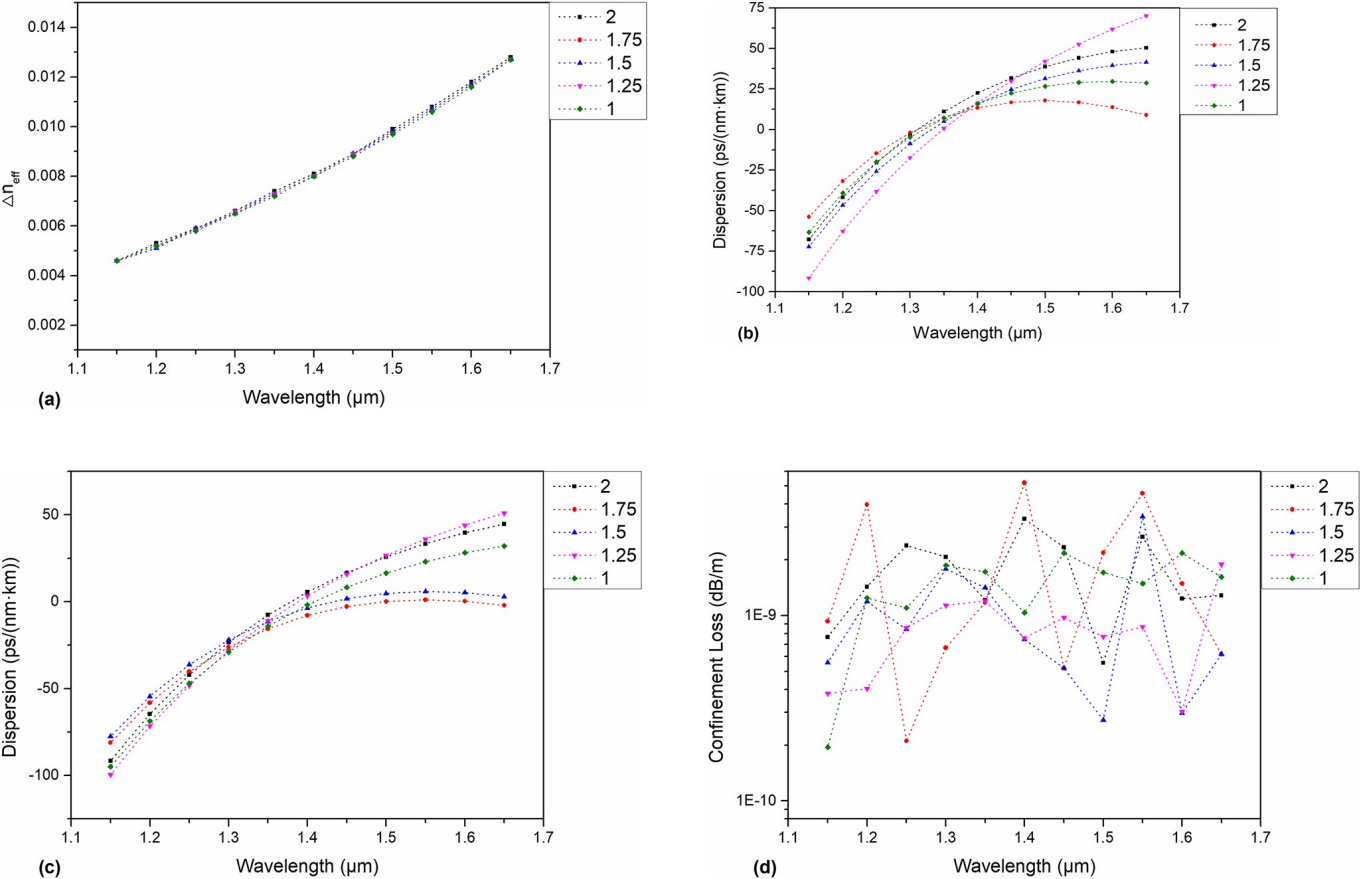
(a) of the HE_3,1_ and EH_1,1_ eigenmodes for a changing long axis: (b) Dispersion of the HE_4,1_ eigenmode for a changing long axis: (c) Dispersion of the TE_0,1_ mode for a changing long axis: (d) Confinement loss of the TE_0,1_ mode for a changing long axis.

As shown in Fig 3(a), there is hardly any influence in the Δn_eff_ between HE_3,1_ and EH_1,1_ for a changing long axis. Therefore, we can conclude that the change in the long axis length during the manufacturing process has little effect on the Δn_eff_. The dispersion curves of the modes HE_4,1_ and TE_0,1_ are shown in Fig 3(b) and 3(c), respectively. It can be seen that the dispersion curve is low and flat when the K value is 1.75, which shows the best performance. As shown in Fig 3(d), the CL is not sensitive to changes in the long axis.

The structure of K = 1.75 in the above comparison is used as the basis to continue the optimization. The long axis of the cladding air holes is set to remain the same, and the length of the short axis changes. The ratio of the short axis to the long axis 1/K changes from 2/7 to 4/7, and the step size is 1/14.

**Fig 4:**
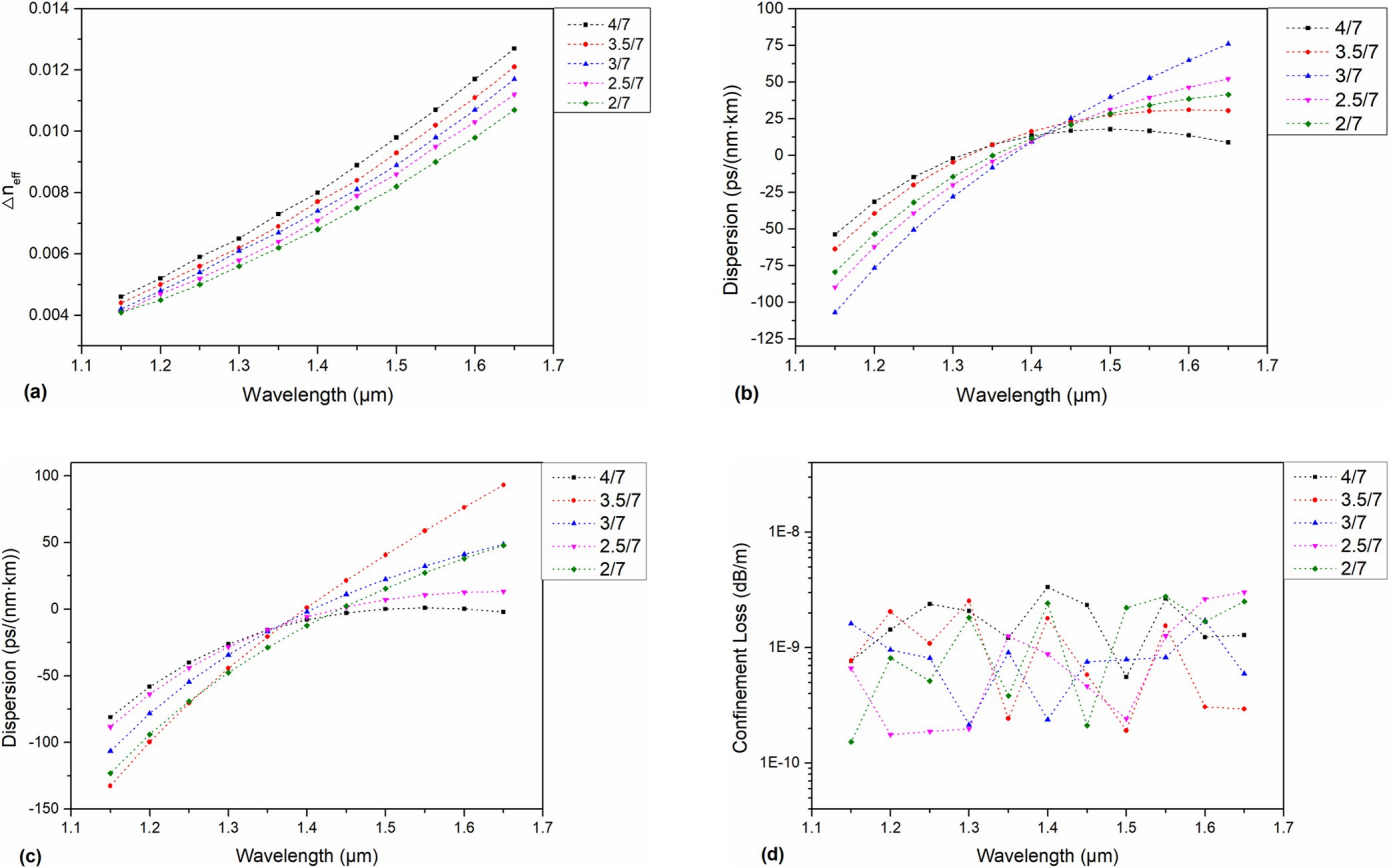
(a) Δn_eff_ of the HE_3,1_ and EH_1,1_ eigenmodes for a changing short axis: (b) Dispersion of the HE_4,1_ eigenmode for a changing short axis: (c) Dispersion of the TE_0,1_ mode for a changing short axis: (d) Confinement loss of the TE_0,1_ mode for a changing short axis.

The Δn_eff_ between EH_1, 1_ and HE_3, 1_ of the eigenmodes is shown in Fig 4(a). It can be seen that the Δn_eff_ increases as the short axis grows until the shortest axis becomes the largest air holes that the structure can accommodate. It can be seen that for a PCF with a semielliptical structure, its Δn_eff_ increases linearly as the minor axis increases. Fig 4(b) and 4(c) respectively show the dispersion curves of modes HE_4,1_ and TE_0,1_ at different 1/K values. When 1/K is 4/7, the dispersion of HE_4,1_ is maintained at 13–18 ps/(nm·km) throughout the C+L band, while the dispersion of TE_0,1_ is basically zero in the C+L band. Fig 4(d) shows the confinement loss of mode TE_0,1_. It can be seen that the change in the 1/K value has little effect on the confinement loss.

### 3.3. Specific performance of the designed structure

**Fig 5:**
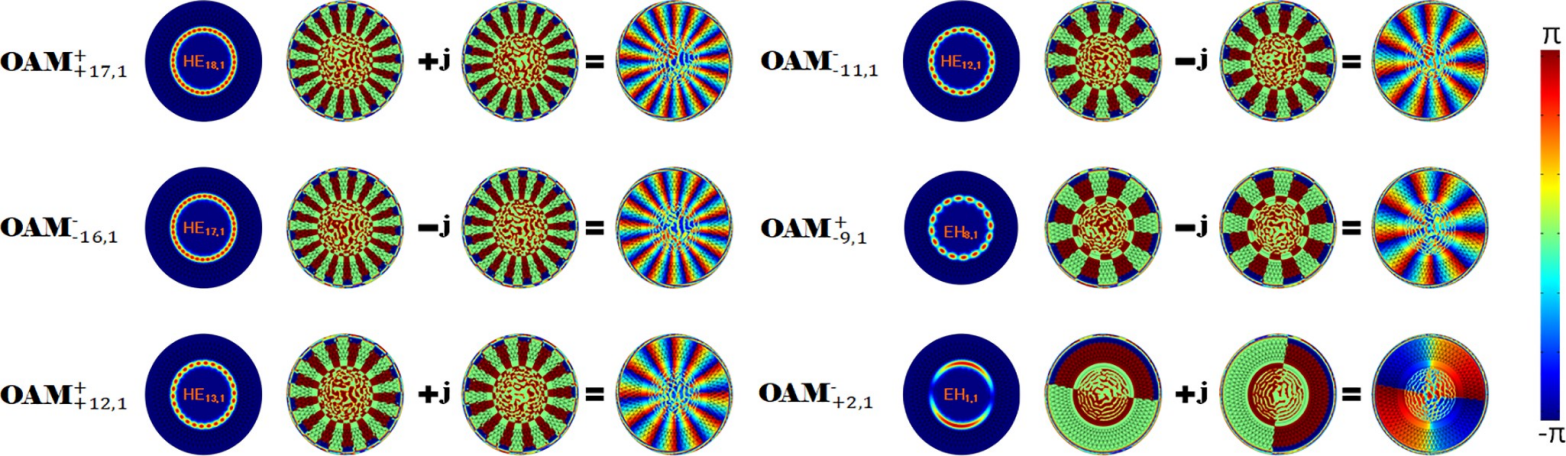
Electric field and phase profiles of the HE_18, 1_, HE_17, 1_, HE_13, 1_, HE_12, 1_, EH_8, 1_ and EH_1, 1_ modes and the phase distributions for the generated OAM_+ +17,1_, OAM_- -16,1_, OAM_+ +12,1_, OAM_- -11,1_, OAM_+ -9,1_, OAM_- +,2,1_ modes.

**Fig 6:**
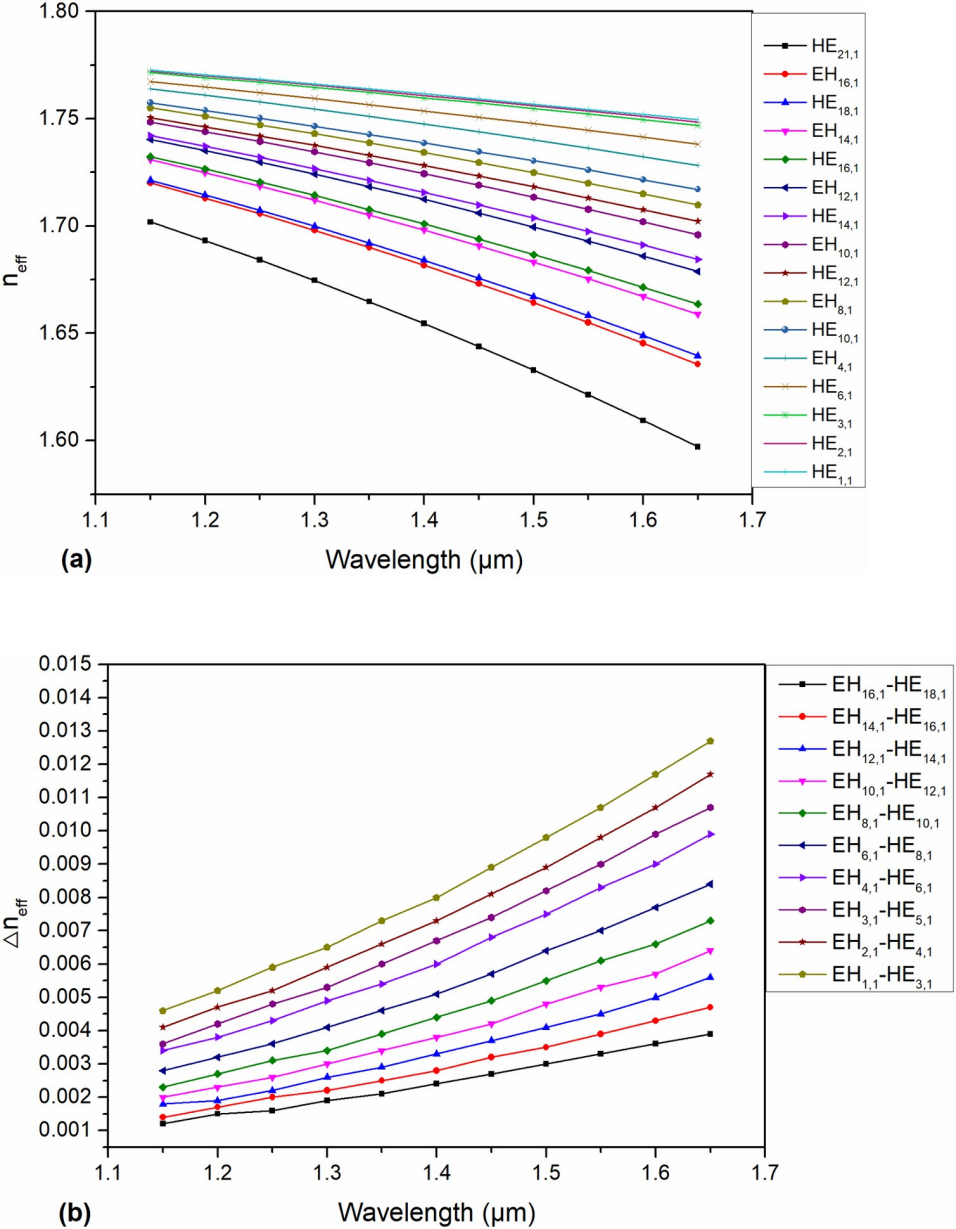
(a) n_eff_ of the eigenmodes guided in the designed PCF as a function of wavelength; (b) Δn_eff_ of the eigenmodes guided in the designed PCF as a function of wavelength.

**Fig 7:**
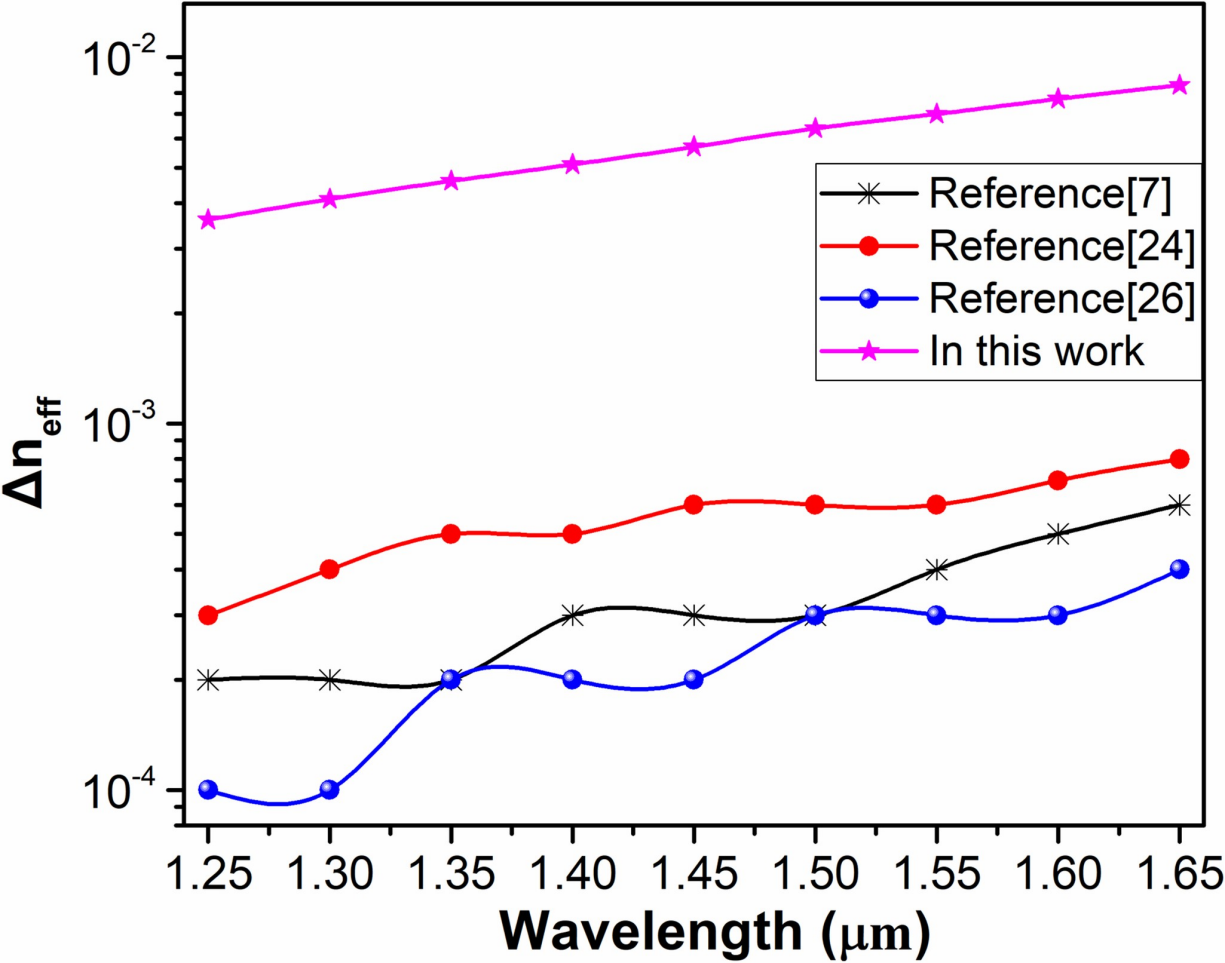
Δn_eff_ of the HE_8,1_ and EH_6,1_ modes guided in the PCF designed in reference [7], [24], [26] and in this paper.

Fig 5 shows the normalized electric field intensity and phase composition of the OAM modes. This figure includes the phase and electric field profiles of the HE_18, 1_, HE_17, 1_, HE_13, 1_, HE_12, 1_, EH_8, 1_ and EH_1, 1_ modes and the phase distributions of the generated OAM modes. The energy of the eigenmode is limited by the cladding, which can reduce energy leakage and is beneficial for long-distance optical communications [30]. Based on couple mode theory, the mode coupling coefficient for two eigenmodes can be described as [31]:

$$\langle P_{m,n}\rangle =\frac{\omega^{2}}{c^{2}}\Phi (\beta_{m}-\beta_{n}){\left(\int \int N_{b}E_{m}^{*}E_{n}rdrd\varphi \right)}^{2},$$ (2)

where

$$\Phi (\beta_{m}-\beta_{n})=\sqrt{\pi }\sigma^{2}\cdot L_{c}\cdot \exp\{-{\left[\frac{1}{2}\left(\beta_{m}-\beta_{n}\right)L_{c}\right]}^{2}\},$$ (3)

$$\beta_{m}-\beta_{n}=\frac{2\pi }{\lambda }\cdot (n_{eff,m}-n_{eff,n})=\frac{2\pi }{\lambda }\cdot \Delta n_{eff},$$ (4)

where ω signifies the angular frequency, and c signifies the velocity of light in vacuum. βm and βn are the adjacent mode’s propagation constants. In addition, Ф(βm-βn) is the spatial power spectrum, which is inversely proportional to Δn_eff_. Nb is connected with the fiber perturbation. σ is the rms-deviation for the deformation in the optical transmission axis, and Lc is the correlation length. It can be concluded that an increase in Δn_eff_ is helpful to avoid coupling between each eigenmode. It was experimentally confirmed that the Δn_eff_ needs to be larger than 10^−4^ to avoid modal coupling [32]. As the number of eigenmodes in the PCF increases, the Δn_eff_ gradually decreases. Therefore, a counterpoise between the quantity of eigenmodes guided in the fiber and a suitable Δn_eff_ should be taken when designing a PCF for high performance. Fig 6 demonstrates that the Δn_eff_ between each eigenmode is above 1×10^−4^, and most values for the Δn_eff_ between the HE_m+1,1_ and EH_m-1,1_ eigenmode sets are greater than 10^−3^; this result means that near-degeneracy into the LP_m,1_ modes will hardly occur and mode crosstalk will almost disappear, remarkably improving the reliability of information transmission. Additionally, Fig 7 suggests a tremendous advance in preventing modal coupling and near-degeneracy compared with our previous work [7] and that of our peers [24,26].

**Fig 8:**
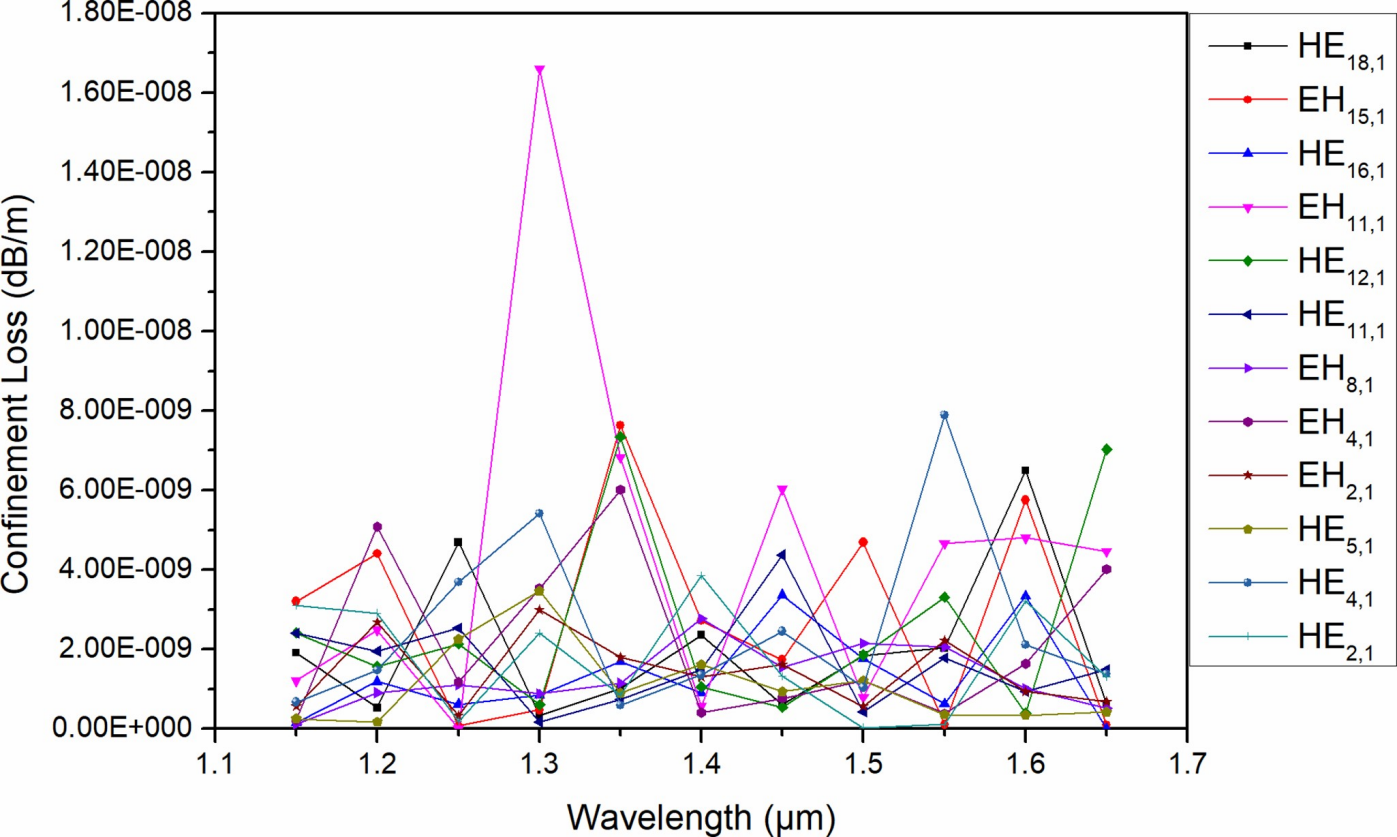
Cl of eigenmodes guided in the designed PCF as a function of wavelength.

The CL for eigenmodes guided in the proposed fiber can be calculated through [7]:

$$L=\frac{2\pi }{\lambda }\frac{20}{\text{ln}(10)}10^{6}Im(n_{eff})(dB/m),$$ (5)

where λ represents the wavelength, and Im(n_eff_) represents the imaginary part of n_eff_ for the eigenmodes. The CL is currently a pivotal factor limiting remote transmission. Thus, we should avoid or minimize the CL. For the purpose of reducing the CL, the air holes in the cladding of the designed PCF are set to five layers, and the light beams are well restrained in the high RI ring-core. As shown in Fig 8, the CL ofthe majority of the eigenmodes in the proposed PCF is less than 10^−8^ dB/m, and the minimum CL is almost as low as 10^−10^ dB/m. There is no doubt that the PCF with a high RI ring-core based on Schott SF57 has superior feasibility, reasonability and reliability.

Due to the great effect of dispersion on optical pulse transmission, the dispersion characteristics of the eigenmodes in this PCF were systematically analyzed. Dispersion in a PCF is dominated by waveguide dispersion and material dispersion, and it is of great significance to study such dispersion. The formula describing waveguide dispersion and material dispersion can be expressed as follows [26]:

$$D_{w}=-\frac{\lambda }{c}\frac{d^{2}n_{eff}}{d\lambda^{2}},$$ (6)

$$D_{m}=-\frac{\lambda }{c}\frac{d^{2}n(\lambda )}{d\lambda^{2}},$$ (7)

$$D\approx D_{w}+D_{m},$$ (8)

where λ denotes the wavelength, and c denotes the speed of light in vacuum.

**Fig 9:**
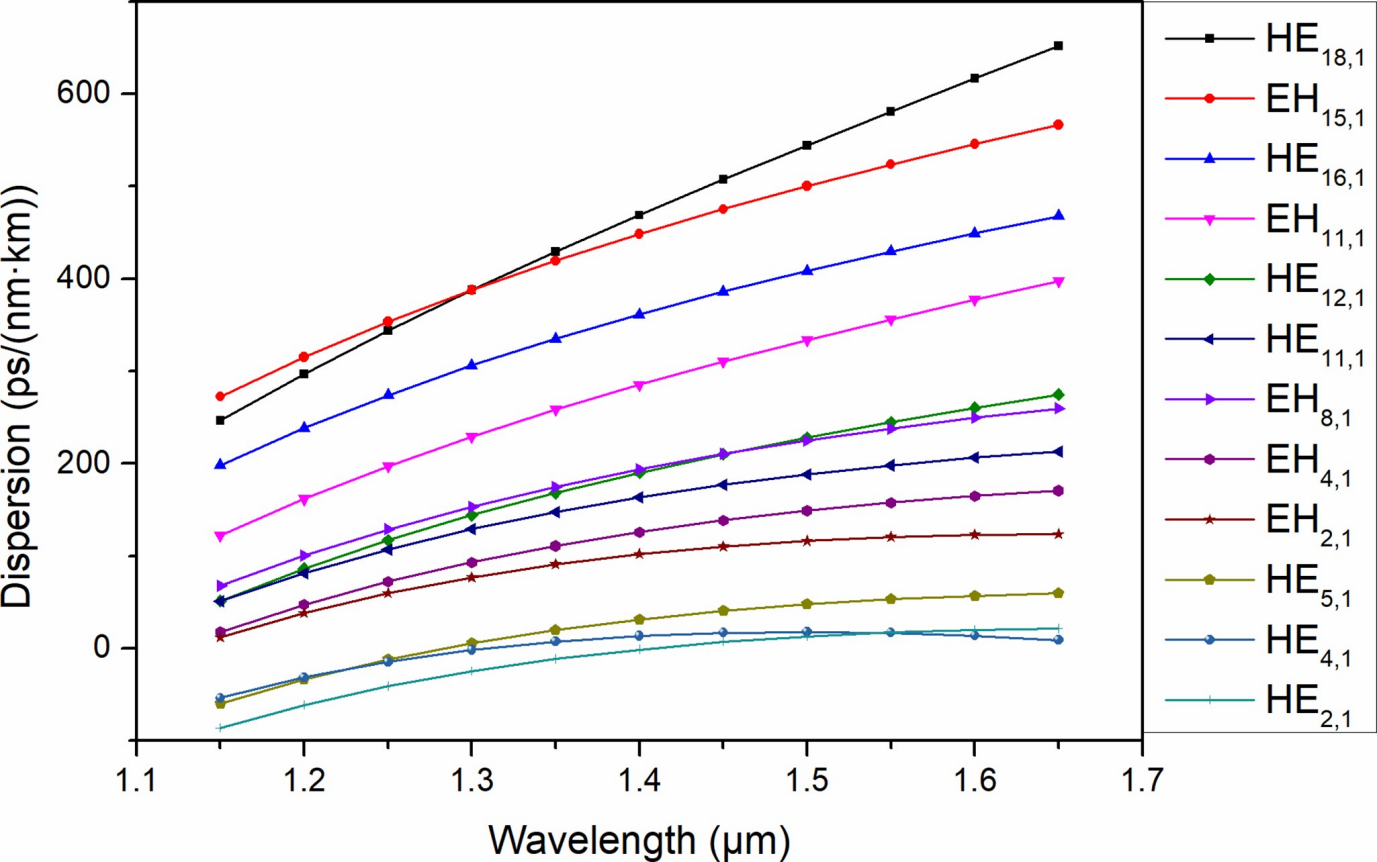
Dispersion of the typical eigenmodes in the proposed PCF as a function of wavelength.

In addition, the Sellmeier equation for Schott SF57 can be described as follows [29]:

$$n^{2}=1+\frac{A_{1}\lambda^{2}}{\lambda^{2}-B_{1}}+\frac{A_{2}\lambda^{2}}{\lambda^{2}-B_{2}}+\frac{A_{3}\lambda^{2}}{\lambda^{2}-B_{3}},$$ (9)

where A_1_ = 1.81651371, A_2_ = 0.428893641, A_3_ = 1.07186278, B_1_ = 0.0143704198, B_2_ = 0.0592801172, and B_3_ = 121.419942. As shown in Fig 9, the simulation results show that the curve for the low mode dispersion is low and flat, especially from 1.4 μm to 1.65 μm, and that the minimum value of chromatic dispersion is as low as 0.105 ps/(nm·km). In practice, the reduction in the value of R_0_ facilitates obtaining a flatter dispersion curve; however, the amount of OAM modes in this fiber is proportional to the size of R_0_. Given the equilibrium between the eigenmode dispersion and amount of OAM modes in this fiber, the structure parameters for this OAM PCF are deemed to be reasonable.

**Fig 10:**
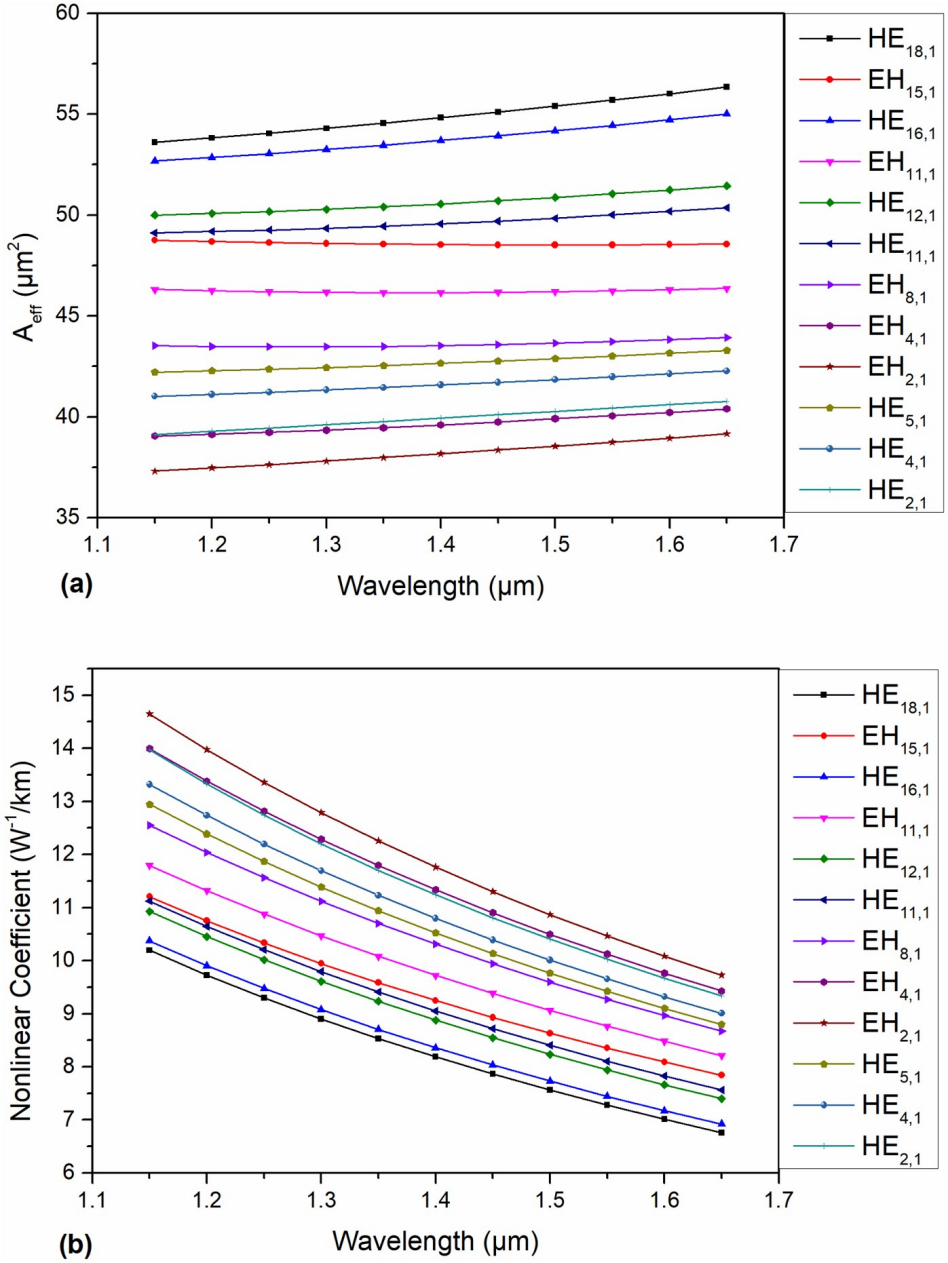
(a) A_eff_ of the typical eigenmodes in the proposed PCF as a function of wavelength; (b) nonlinear coefficient of the typical eigenmodes in the proposed PCF as a function of wavelength.

In addition, we also carried out a numerical analysis of the effective mode area (A_eff_) and the nonlinear coefficient of the eigenmode. The A_eff_ is given by [33]:

$$A_{eff}={\left[\int_{0}^{2\pi }\int_{0}^{\infty }u{\left(\rho ,\varphi \right)}^{2}\rho d\rho d\varphi \right]}^{2}/\int_{0}^{2\pi }\int_{0}^{\infty }u{\left(\rho ,\varphi \right)}^{4}\rho d\rho d\varphi ,$$ (10)

where u(ρ,φ) denotes the electric field distributions of the eigenmodes. The nonlinear coefficient can be calculated from the following equation [34]:

$$\gamma =\frac{2\pi n_{2}}{\lambda A_{eff}},$$ (11)

where n_2_ = 10^−19^ m^2^w^-1^ is the nonlinear RI for Schott SF57. A_eff_ and nonlinear coefficients of the eigenmodes are shown in Fig 10(a) and 10(b), respectively. The curve of A_eff_ is gentle, and all the eigenmodes guided in this PCF possess much higher values of A_eff_ than those in a conventional fiber. It is worth noting that the maximum A_eff_ of all the modes is 57.8 μm^2^, which is considerable. The nonlinear coefficients range from 6.31 w^-1^/km to 14.8 w^-1^/km, which is significantly less than that in reference [26]. In addition, the minimum nonlinear coefficient of the TE_0,1_ mode is 6.31 w^-1^/km at 1.65 μm.

## 4. Results and discussion

In summary, we optimized a PCF structure from the literature [25] and developed a PCF structure composed of a high RI ring-core and 5 well-ordered semielliptical air holes. A comprehensive study of the eigenmode properties was carried out in this paper. First, taking the structure from reference [26] as an example, the structures with and without a ring-core were compared. When the substrate materials of the cladding are the same, the comparison results show that a PCF with a ring-core can work better than a PCF without a ring-core. Then, we compared the long axis and the short axis of the changing cladding air holes separately, and we selected the optimal results. The Δn_eff_ increased linearly with increasing long axis of the air holes, while the short axis showed no effect on the Δn_eff_. Setting the long axis of the air holes to be as long as possible is helpful for increasing the Δn_eff_. The Δn_eff_ value between the HE_m+1, 1_ mode and EH_m-1, 1_ (m = 2–20) reaches up to 10^−3^ and is larger than the previously described values, which means the modal coupling can be almost neglected. The dispersion curve of the low order modes is low and flat, especially at the C+L band, and the minimum value for the chromatic dispersion is as low as 0.105 ps/(nm·km). The TE_0,1_ mode has a 200 nm ZDM and has potential applications in the direction of supercontinuum generation [35]. In addition, there is practical possibility to change the ellipticity of the air holes [36]. The PCF structure with high RI ring core and semi-ellipsoidal air holes has mature conditions for manufacturing, and has experimental significance for optical communication and supercontinuum generation [37][38].

## Supporting information

S1 Table Detailed data of the optimal structure, including Δn_eff_, dispersion, confinement loss, etc. (ZIP)

S2 Table Detailed data when changing the long axis and short axis, including Δn_eff_, dispersion, confinement loss, etc. (ZIP)
