# Supplementary material for: A Photonic crystal fiber with large effective refractive index separation and low dispersion
Source: PLoS One. 2020 May 14;15(5):e0232982. doi: 10.1371/journal.pone.0232982 (PMC7224559; doi:10.1371/journal.pone.0232982)
Supplement: S1 Table — (ZIP) [file pone.0232982.s001.zip › S1 Table/Effective Refractive Index Separation.pdf]

	EH18-HE2	EH17-HE1	EH16-HE1	EH15-HE1	EH14-HE1	EH13-HE1	EH12-HE1	EH11-HE1
1.15	0.0011	0.0011	0.0012	0.0014	0.0014	0.0016	0.0018	0.0019
1.2	0.0012	0.0013	0.0015	0.0015	0.0017	0.0018	0.0019	0.0022
1.25	0.0013	0.0015	0.0016	0.0017	0.002	0.0021	0.0022	0.0025
1.3	0.0016	0.0017	0.0019	0.002	0.0022	0.0024	0.0026	0.0028
1.35	0.0018	0.002	0.0021	0.0023	0.0025	0.0027	0.0029	0.0031
1.4	0.0019	0.0021	0.0024	0.0026	0.0028	0.0031	0.0033	0.0035
1.45	0.0022	0.0024	0.0027	0.0029	0.0032	0.0034	0.0037	0.0039
1.5	0.0024	0.0027	0.003	0.0033	0.0035	0.0038	0.0041	0.0044
1.55	0.0026	0.003	0.0033	0.0036	0.0039	0.0042	0.0045	0.0049
1.6	0.0028	0.0032	0.0036	0.004	0.0043	0.0047	0.005	0.0054
1.65	0.0031	0.0035	0.0039	0.0044	0.0047	0.0051	0.0056	0.0059

EH10-HE1	EH9-HE11	EH8-HE1C	EH7-HE9	EH6-HE8	EH5-HE7	EH4-HE6	EH3-HE5	EH2-HE4
0.002	0.0021	0.0023	0.0025	0.0028	0.0031	0.0034	0.0036	0.0041
0.0023	0.0025	0.0027	0.0029	0.0032	0.0035	0.0038	0.0042	0.0047
0.0026	0.0029	0.0031	0.0033	0.0036	0.0039	0.0043	0.0048	0.0052
0.003	0.0032	0.0034	0.0038	0.0041	0.0045	0.0049	0.0053	0.0059
0.0034	0.0037	0.0039	0.0042	0.0046	0.0049	0.0054	0.006	0.0066
0.0038	0.0041	0.0044	0.0048	0.0051	0.0055	0.006	0.0067	0.0073
0.0042	0.0045	0.0049	0.0053	0.0057	0.0062	0.0068	0.0074	0.0081
0.0048	0.005	0.0055	0.0059	0.0064	0.0069	0.0075	0.0082	0.0089
0.0053	0.0056	0.0061	0.0065	0.007	0.0075	0.0083	0.009	0.0098
0.0057	0.0062	0.0066	0.0072	0.0077	0.0083	0.009	0.0099	0.0107
0.0064	0.0068	0.0073	0.0078	0.0084	0.0092	0.0099	0.0107	0.0117

EH1-HE3
0.0046
0.0052
0.0059
0.0065
0.0073
0.008
0.0089
0.0098
0.0107
0.0117
0.0127
