# Supplementary material for: A Photonic crystal fiber with large effective refractive index separation and low dispersion
Source: PLoS One. 2020 May 14;15(5):e0232982. doi: 10.1371/journal.pone.0232982 (PMC7224559; doi:10.1371/journal.pone.0232982)
Supplement: S1 Table — (ZIP) [file pone.0232982.s001.zip › S1 Table/nonlinear coefficient.pdf]

	HE21,1	EH18,1	HE20,1	EH17,1	HE19,1	EH16,1	HE18,1	EH15,1
1.15	10.00610966	10.97413	10.05976	11.03673	10.12111	11.11677	10.19437	11.20845
1.2	9.542196352	10.53021	9.595192	10.58982	9.656174	10.66683	9.729547	10.75539
1.25	9.113007452	10.1209	9.166595	10.17748	9.227054	10.25144	9.300534	10.33612
1.3	8.716001141	9.742507	8.768949	9.795948	8.829423	9.86648	8.902426	9.947992
1.35	8.345867262	9.390471	8.399768	9.440974	8.45997	9.507853	8.532501	9.585373
1.4	7.972178538	9.063014	8.055628	9.110529	8.1153	9.173416	8.187285	9.247451
1.45	7.683667504	8.756493	7.734738	8.801101	7.793567	8.859964	7.86434	8.931083
1.5	7.3843568	8.469327	7.434442	8.511545	7.492665	8.567014	7.561502	8.633636
1.55	7.103523735	8.200157	7.152366	8.239199	7.209573	8.291277	7.27811	8.353947
1.6	6.840179645	7.947663	6.887275	7.982581	6.943346	8.031495	7.010864	8.090348
1.65	6.592614634	7.708695	6.638199	7.74124	6.692337	7.786049	6.759019	7.841965

HE17,1	EH14,1	HE16,1	EH13,1	HE15,1	EH12,1	HE14,1	EH11,1	HE13,1
10.27649	11.3232	10.37222	11.45444	10.48367	11.61489	10.61017	11.79704	10.75923
9.811698	10.86636	9.906377	10.99237	10.01822	11.14634	10.14349	11.32152	10.29004
9.381809	10.44264	9.477002	10.56451	9.587039	10.71226	9.710851	10.8801	9.855355
8.983669	10.04946	9.077594	10.16592	9.186335	10.30808	9.309136	10.46866	9.451845
8.613275	9.683142	8.706251	9.794619	8.813465	9.929942	8.935604	10.08354	9.07594
8.267271	9.340432	8.358945	9.447121	8.466338	9.577063	8.585894	9.723215	8.724367
7.943405	9.019395	8.035936	9.121286	8.140288	9.245054	8.25917	9.385326	8.395033
7.639827	8.718337	7.73201	8.814492	7.835041	8.933141	7.951571	9.066377	8.085857
7.355096	8.434115	7.446151	8.526427	7.54768	8.638705	7.662658	8.766951	7.794705
7.087487	8.165908	7.176695	8.254412	7.276984	8.361193	7.390786	8.482347	7.520261
6.833785	7.912843	6.922125	7.996747	7.021402	8.098632	7.133421	8.213595	7.260631

EH10,1	HE12,1	EH9,1	HE11,1	EH8,1	HE10,1	EH7,1	EH6,1	HE9,1
12.01618	10.92691	12.26343	11.12331	12.55304	11.34511	12.8727	13.23204	11.60294
11.53147	10.45501	11.767	10.64743	12.04078	10.8631	12.34248	12.67834	11.11268
11.08033	10.01748	11.30402	10.20622	11.56483	10.41634	11.84816	12.16235	10.65785
10.65907	9.611237	10.87286	9.795575	11.11941	10.00042	11.38668	11.68192	10.2342
10.26604	9.232409	10.46961	9.412534	10.70322	9.612598	10.95499	11.23222	9.839091
9.897836	8.878434	10.09056	9.054543	10.31247	9.249545	10.55082	10.81	9.469597
9.551117	8.546849	9.734365	8.718721	9.944487	8.909097	10.17015	10.41432	9.122568
9.224394	8.234797	9.39948	8.403713	9.59875	8.589446	9.811039	10.04188	8.796996
8.916211	7.940649	9.082988	8.106796	9.272427	8.288189	9.473804	9.690869	8.490163
8.625239	7.663556	8.78403	7.826141	8.962934	8.003671	9.154251	9.358901	8.200368
8.350293	7.401965	8.500666	7.561707	8.670666	7.734959	8.852025	9.045858	7.926561

EH5,1	HE8,1	EH4,1	EH3,1	HE7,1	EH2,1	EH1,1	TM0,1	HE6,1
13.60652	11.88994	13.99252	14.3467	12.21595	14.64537	14.80682	9.944992	12.56818
13.02565	11.38877	13.38155	13.70941	11.69963	13.97653	14.11928	9.480458	12.0333
12.48634	10.92334	12.81458	13.11472	11.22093	13.35843	13.48668	9.05366	11.53691
11.98325	10.4912	12.28674	12.56405	10.7745	12.78666	12.90127	8.658941	11.07552
11.51318	10.08651	11.7954	12.05052	10.35888	12.25496	12.35889	8.292848	10.6447
11.0733	9.708291	11.33556	11.57275	9.969487	11.76071	11.85386	7.952975	10.2428
10.66067	9.353672	10.90458	11.12594	9.605042	11.29925	11.38426	7.636429	9.866293
10.27355	9.022884	10.49538	10.70662	9.262444	10.86711	10.94429	7.341346	9.513073
9.90841	8.706624	10.12175	10.31364	8.940081	10.46327	10.53347	7.064812	9.181328
9.563982	8.410873	9.7645	9.944564	8.636361	10.0833	10.14679	6.804861	8.868413
9.23876	8.131156	9.427449	9.595946	8.349398	9.726145	9.783814	6.561665	8.573037

HE5,1	HE4,1	HE3,1	HE2,1	HE1,1	TE0,1
12.94394	13.32039	13.67674	13.96792	14.12631	9.488433
12.38654	12.73647	13.06461	13.33073	13.47446	9.047891
11.86885	12.19525	12.50043	12.74479	12.87572	8.643553
11.38839	11.69505	11.97765	12.20352	12.32353	8.270986
10.94142	11.22909	11.49347	11.70307	11.81312	7.927165
10.52453	10.79509	11.04319	11.23849	11.34026	7.608763
10.13398	10.39025	10.62311	10.8065	10.9004	7.311957
9.767083	10.01137	10.23098	10.40347	10.49023	7.036223
9.42476	9.655493	9.864862	10.02623	10.10849	6.779695
9.101042	9.322356	9.520192	9.673594	9.751982	6.539403
8.797356	9.008576	9.197436	9.342655	9.4179	6.313862
