# Supplementary material for: A Photonic crystal fiber with large effective refractive index separation and low dispersion
Source: PLoS One. 2020 May 14;15(5):e0232982. doi: 10.1371/journal.pone.0232982 (PMC7224559; doi:10.1371/journal.pone.0232982)
Supplement: S1 Table — (ZIP) [file pone.0232982.s001.zip › S1 Table/SF57-confinement loss.pdf]

	HE21,1	EH18,1	HE20,1	EH17,1	HE19,1	EH16,1	HE18,1	EH15,1	HE17,1
1.15	1.15E-05	8.39E-09	3.43E-09	7.55E-10	3.44E-09	2.3E-09	1.92E-09	3.2E-09	7.83E-09
1.2	1.81E-05	3.01E-10	4.02E-09	2.04E-09	4.5E-10	4.69E-09	5.32E-10	4.4E-09	1.41E-09
1.25	0.000441	3.78E-09	2.75E-09	1.05E-08	2.71E-09	7.4E-11	4.69E-09	7.23E-11	3.03E-09
1.3	0.000909	5.75E-09	6.31E-09	2.85E-09	4.32E-09	1.18E-09	3.46E-10	4.76E-10	7.29E-09
1.35	0.002057	4.5E-09	9.67E-10	2.81E-09	3.13E-09	4.04E-09	1.01E-09	7.63E-09	2.76E-09
1.4	0.0003	5.48E-09	8.72E-11	3.45E-10	6.51E-09	3.97E-09	2.36E-09	2.72E-09	5.02E-09
1.45	6.84E-10	7.83E-09	6.29E-09	1.64E-09	1.47E-09	7.4E-10	6.04E-10	1.74E-09	2.38E-09
1.5	3.11E-09	9.13E-10	8.08E-09	1.51E-09	7.66E-09	3.63E-09	1.84E-09	4.69E-09	1.33E-09
1.55	8.89E-09	6.27E-09	1.01E-09	8.81E-10	5.81E-09	3.22E-09	2.03E-09	1.07E-10	3.74E-09
1.6	3.77E-07	9.71E-09	3.41E-10	2.65E-09	4.36E-09	3.49E-09	6.5E-09	5.76E-09	3.64E-09
1.65	5.73E-05	2.44E-09	1.23E-08	9.61E-09	9.28E-10	3.63E-09	6.29E-10	9.84E-11	1.23E-09

EH14,1	HE16,1	EH13,1	HE15,1	EH12,1	HE14,1	EH11,1	HE13,1	EH10,1	HE12,1
1.73E-09	1.54E-10	2.29E-09	2.28E-09	3.52E-09	1.1E-09	1.21E-09	3.94E-10	1.06E-09	2.42E-09
4.36E-09	1.19E-09	4E-09	1.58E-09	3.42E-09	2.02E-09	2.49E-09	1.35E-09	3.67E-09	1.56E-09
1.16E-09	6.19E-10	5.62E-09	6.26E-09	5.22E-12	1.01E-09	1.04E-11	2.81E-09	1.17E-09	2.14E-09
2.31E-09	8.42E-10	1.81E-09	5.37E-11	1.37E-09	3.42E-09	1.66E-08	3.38E-09	2.43E-09	6.05E-10
9.07E-10	1.7E-09	3.66E-09	5.73E-09	4.06E-09	5.25E-09	6.83E-09	2.33E-09	9.43E-10	7.35E-09
3.47E-09	8.97E-10	2.6E-09	3.48E-09	9.11E-10	1.73E-09	5.64E-10	1.66E-09	9.23E-10	1.06E-09
2.99E-09	3.37E-09	5.13E-10	3.66E-09	8.84E-10	5.67E-09	6.03E-09	2.92E-09	1.13E-09	5.44E-10
1.21E-09	1.77E-09	4.6E-09	8.28E-10	6.72E-09	6.3E-09	7.91E-10	2.55E-09	4.44E-09	1.86E-09
2.76E-10	6.22E-10	1.72E-09	7.86E-10	2.19E-09	2.42E-09	4.66E-09	2.04E-09	5.92E-09	3.31E-09
4.49E-09	3.35E-09	2.95E-09	1.12E-09	3E-09	2.02E-09	4.8E-09	7.43E-09	6.5E-09	4E-10
1.05E-08	1.16E-12	1.4E-09	3.79E-09	1.17E-08	1.83E-09	4.46E-09	1.11E-08	1.39E-09	7.03E-09

EH9,1	HE11,1	EH8,1	HE10,1	EH7,1	EH6,1	HE9,1	EH5,1	HE8,1	EH4,1
4.38E-10	2.4E-09	1.13E-10	2.01E-09	1.68E-09	7.19E-10	8.93E-10	1.34E-10	1.72E-10	2.25E-10
2.82E-09	1.96E-09	9.1E-10	2.49E-09	5.73E-10	1.44E-09	2.36E-10	9.05E-10	4.98E-09	5.09E-09
1.43E-09	2.54E-09	1.1E-09	2.66E-09	1.75E-09	1.89E-12	3.66E-10	4.12E-09	8.02E-10	1.18E-09
1.31E-09	1.79E-10	8.83E-10	6.92E-10	1.21E-09	2.25E-09	1.69E-09	3.67E-09	9.21E-10	3.53E-09
3.11E-09	7.42E-10	1.15E-09	8.75E-09	3.28E-09	1.37E-09	7.9E-10	1.85E-09	6.99E-11	6.01E-09
3.32E-10	1.49E-09	2.77E-09	2.83E-09	2.56E-09	2.03E-09	2.87E-09	2.52E-09	3.91E-10	4.11E-10
7.22E-09	4.38E-09	1.55E-09	4.08E-09	3.31E-09	4.3E-10	2.6E-09	1.87E-09	2.91E-09	7.61E-10
3.33E-09	4.22E-10	2.15E-09	2.58E-09	2.95E-09	1.4E-09	3.45E-09	3.8E-09	6.86E-10	1.21E-09
1.35E-09	1.79E-09	2.06E-09	5.19E-10	3.92E-10	3.95E-09	4.29E-10	2.98E-09	1.46E-09	3.8E-10
2.6E-09	9.44E-10	1.02E-09	3.12E-10	1.58E-09	5.19E-09	6.86E-10	1.56E-09	7.48E-09	1.64E-09
1.36E-08	1.49E-09	5.16E-10	2.11E-09	2.06E-09	3.05E-09	2.16E-09	1.4E-09	2.14E-10	4.01E-09

EH3,1	HE7,1	EH2,1	EH1,1	TM0,1	HE6,1	HE5,1	HE4,1	HE3,1	HE2,1
2E-09	3.17E-11	5.67E-10	2.52E-10	2.57E-09	9.17E-10	2.51E-10	6.73E-10	2.89E-10	3.1E-09
3.07E-10	1.53E-09	2.68E-09	1.47E-09	8.22E-10	2.41E-09	1.75E-10	1.47E-09	1.45E-09	2.91E-09
1.9E-10	6.92E-10	3.35E-10	1.3E-09	1.74E-09	9.19E-10	2.25E-09	3.69E-09	1.09E-09	1.93E-10
1.77E-10	8.91E-11	2.99E-09	1.05E-09	1.72E-09	2.9E-09	3.46E-09	5.41E-09	4.08E-10	2.41E-09
1.78E-09	1.35E-09	1.8E-09	1.85E-09	1.1E-09	4.3E-09	9.01E-10	5.96E-10	1.63E-09	8.43E-10
1.27E-09	1.86E-09	1.31E-09	1.59E-09	1.25E-09	1.5E-09	1.61E-09	1.36E-09	6.07E-10	3.85E-09
1.86E-09	1.32E-09	1.61E-09	2.53E-09	1.74E-09	2.84E-09	9.37E-10	2.45E-09	1.38E-09	1.32E-09
6.43E-10	1.82E-09	5.67E-10	1.12E-09	3.99E-10	6.97E-10	1.21E-09	1.04E-09	1.63E-09	3.33E-11
1.48E-09	3.03E-12	2.22E-09	1.67E-09	1.74E-09	3.28E-09	3.6E-10	7.9E-09	3.98E-09	1.1E-10
4.35E-09	2.83E-10	9.37E-10	1.07E-12	9.31E-10	2.47E-09	3.43E-10	2.12E-09	7.56E-10	3.22E-09
2.73E-09	2.51E-09	6.84E-10	7.99E-10	2.77E-10	1.86E-09	4.2E-10	1.38E-09	3.71E-09	1.3E-09

HE1,1	TE0,1
6.58E-11	9.31E-10
2.59E-09	3.97E-09
9.94E-10	2.11E-10
1.04E-09	6.7E-10
6.55E-10	1.18E-09
2.72E-09	5.21E-09
1.56E-09	5.21E-10
2.78E-09	2.18E-09
2.3E-09	4.57E-09
2.41E-09	1.49E-09
1.57E-09	6.2E-10
