# Supplementary material for: A Photonic crystal fiber with large effective refractive index separation and low dispersion
Source: PLoS One. 2020 May 14;15(5):e0232982. doi: 10.1371/journal.pone.0232982 (PMC7224559; doi:10.1371/journal.pone.0232982)
Supplement: S1 Table — (ZIP) [file pone.0232982.s001.zip › S1 Table/SF57-dispersion.pdf]

	HE21,1	EH18,1	HE20,1	EH17,1	HE19,1	EH16,1	HE18,1	EH15,1	HE17,1
**1.15**	428.156	364.355	330.045	329.338	304.31	290.37	246.23	272.321	190.648
**1.2**	483.696	421.037	389.337	380.766	356.516	337.871	296.843	315.118	239.405
**1.25**	535.209	474.019	445.271	428.183	404.866	381.174	343.678	353.39	284.43
**1.3**	583.529	524.134	498.68	472.422	450.195	421.114	387.569	387.973	326.558
**1.35**	629.299	572.026	550.208	514.127	493.145	458.334	429.16	419.509	366.432
**1.4**	673.021	618.196	600.356	553.799	534.218	493.334	468.95	448.499	404.552
**1.45**	715.089	663.038	649.518	591.832	573.807	526.51	507.334	475.338	441.313
**1.5**	755.816	706.866	698.007	628.54	612.227	558.175	544.627	500.339	477.03
**1.55**	795.455	749.932	746.076	664.175	649.729	588.58	581.081	523.754	511.953
**1.6**	834.209	792.44	793.929	698.94	686.518	617.931	616.898	545.787	546.287
**1.65**	872.246	834.556	841.732	733.004	722.759	646.392	652.246	566.605	580.198

EH14,1	HE16,1	EH13,1	HE15,1	EH12,1	HE14,1	EH11,1	HE13,1	EH10,1	HE12,1
210.189	198.155	176.327	134.901	145.183	90.395	122.119	72.171	100.758	51.089
254.375	238.287	219.245	177.129	186.385	130.877	161.759	109.622	137.215	86.13
294.377	273.941	257.997	215.284	223.39	167.3	197.156	142.827	169.24	116.801
331.03	305.952	293.416	250.199	257.032	200.499	229.145	172.622	197.669	143.938
364.977	334.963	326.146	282.518	287.956	231.118	258.368	199.65	223.145	168.183
396.719	361.475	356.688	312.741	316.661	259.657	285.326	224.412	246.168	190.038
426.65	385.882	385.437	341.264	343.542	286.511	310.414	247.302	267.133	209.896
455.084	408.497	412.706	368.4	368.914	311.993	333.945	268.633	286.353	228.072
482.274	429.574	438.747	394.4	393.027	336.356	356.172	288.659	304.081	244.818
508.422	449.315	463.764	419.469	416.087	359.803	377.298	307.583	320.52	260.337
533.697	467.888	487.923	443.773	438.258	382.501	397.49	325.57	335.837	274.795

EH9,1	HE11,1	EH8,1	HE10,1	EH7,1	EH6,1	HE9,1	EH5,1	HE8,1	EH4,1
37.139	50.719	67.343	2.109	27.132	23.578	-29.498	4.462	-52.75	17.834
79.78	81.27	100.295	35.396	61.506	55.928	4.09	39.527	-19.798	47.33
118.737	107.08	128.645	64.344	91.542	83.785	33.48	70.393	8.986	72.114
154.842	128.982	153.227	89.79	118.075	107.983	59.506	97.896	34.439	93.022
188.74	147.621	174.685	112.375	141.747	129.165	82.811	122.679	57.202	110.697
220.931	163.495	193.519	132.601	163.06	147.831	103.897	145.241	77.776	125.64
251.81	177.001	210.123	150.862	182.408	164.377	123.157	165.978	96.556	138.244
281.69	188.451	224.813	167.472	200.104	179.115	140.906	185.204	113.856	148.825
310.824	198.098	237.839	182.683	216.402	192.299	157.395	203.171	129.928	157.633
339.415	206.146	249.405	196.698	231.504	204.132	172.829	220.081	144.975	164.874
367.63	212.761	259.678	209.685	245.577	214.78	187.373	236.103	159.165	170.712

EH3,1	HE7,1	EH2,1	EH1,1	TM0,1	HE6,1	HE5,1	HE4,1	HE3,1	HE2,1
-0.929	-54.898	11.582	-30.416	-38.818	-84.021	-59.724	-53.827	-103.506	-86.53
27.937	-25.395	38.008	2.203	-6.751	-52.243	-33.601	-31.736	-74.252	-61.572
52.107	-0.34	59.489	30.545	21.025	-24.617	-12.189	-14.715	-49.29	-41.326
72.417	21.104	76.862	55.447	45.345	-0.307	5.346	-1.926	-27.784	-24.956
89.509	39.579	90.768	77.551	66.851	21.329	19.649	7.271	-9.093	-11.82
103.884	55.585	101.708	97.357	86.044	40.791	31.219	13.379	7.286	-1.415
115.936	69.518	110.077	115.261	103.319	58.475	40.45	16.791	21.747	6.651
125.98	81.691	116.19	131.575	118.99	74.694	47.658	17.822	34.603	12.693
134.268	92.355	120.296	146.553	133.308	89.701	53.094	16.723	46.107	16.963
141.002	101.716	122.602	160.397	146.477	103.698	56.961	13.698	56.461	19.665
146.35	109.939	123.272	173.275	158.664	116.853	59.427	8.914	65.834	20.965

HE1,1	TE0,1
-107.977	-81.167
-80.222	-58.214
-56.867	-40.16
-37.078	-26.168
-20.212	-15.595
-5.767	-7.942
6.651	-2.813
17.356	0.105
26.599	1.064
34.585	0.269
41.48	-2.115
