# Supplementary material for: A Photonic crystal fiber with large effective refractive index separation and low dispersion
Source: PLoS One. 2020 May 14;15(5):e0232982. doi: 10.1371/journal.pone.0232982 (PMC7224559; doi:10.1371/journal.pone.0232982)
Supplement: S1 Table — (ZIP) [file pone.0232982.s001.zip › S1 Table/the effective mode area.pdf]

	HE21,1	EH18,1	HE20,1	EH17,1	HE19,1	EH16,1	HE18,1	EH15,1
1.15	54.60303	49.78653	54.3118	49.50415	53.98262	49.14772	53.5947	48.74573
1.2	54.87193	49.7235	54.56887	49.44359	54.22425	49.08662	53.81533	48.68243
1.25	55.15795	49.66505	54.8355	49.38891	54.4762	49.0326	54.0458	48.63091
1.3	55.45226	49.60961	55.11743	49.33897	54.73992	48.98626	54.29104	48.58487
1.35	55.76666	49.56313	55.40881	49.298	55.01451	48.95123	54.54686	48.55535
1.4	56.29565	49.51984	55.71247	49.26157	55.30282	48.92386	54.81658	48.53218
1.45	56.39535	49.48592	56.02299	49.2351	55.6001	48.908	55.09974	48.51854
1.5	56.72519	49.45836	56.34304	49.21304	55.90521	48.8944	55.39627	48.5171
1.55	57.06559	49.43403	56.6759	49.19978	56.22619	48.89076	55.69671	48.52398
1.6	57.41064	49.41064	57.01806	49.1945	56.55761	48.89489	56.01294	48.53921
1.65	57.76147	49.39865	57.36482	49.19097	56.90077	48.90787	56.33941	48.55915

HE17,1	EH14,1	HE16,1	EH13,1	HE15,1	EH12,1	HE14,1	EH11,1	HE13,1
53.16641	48.25172	52.67572	47.69886	52.11573	47.03995	51.49438	46.31365	50.78093
53.36475	48.18529	52.85472	47.63294	52.26463	46.97494	51.61917	46.24811	50.88404
53.5776	48.13482	53.03944	47.57956	52.43067	46.92332	51.76218	46.19945	51.00322
53.80006	48.0943	53.2434	47.54334	52.61314	46.88768	51.9191	46.16847	51.1352
54.03533	48.06509	53.45827	47.51805	52.80797	46.87048	52.08614	46.15652	51.28076
54.28623	48.04906	53.69086	47.50642	53.00981	46.86185	52.27166	46.15747	51.44201
54.55131	48.04348	53.92317	47.5068	53.23192	46.8708	52.4657	46.17028	51.61661
54.82833	48.04575	54.17466	47.52163	53.46226	46.89045	52.67877	46.20137	51.80391
55.11373	48.06276	54.43977	47.5424	53.70747	46.92449	52.90159	46.23806	52.0054
55.40738	48.09007	54.71866	47.57444	53.96454	46.96687	53.1336	46.29604	52.21881
55.72302	48.12418	55.01188	47.61925	54.23406	47.02018	53.3824	46.36205	52.44711

EH10,1	HE12,1	EH9,1	HE11,1	EH8,1	HE10,1	EH7,1	EH6,1	HE9,1
45.46904	50.00171	44.5523	49.11882	43.52442	48.15855	42.44363	41.291	47.08839
45.40609	50.08116	44.49721	49.17608	43.48545	48.19976	42.4225	41.29868	47.11725
45.36461	50.17777	44.46692	49.24987	43.46409	48.25639	42.42473	41.32875	47.16287
45.34371	50.28717	44.45214	49.34084	43.46652	48.33019	42.44626	41.37349	47.22618
45.33601	50.41167	44.45448	49.44695	43.48423	48.41783	42.48484	41.43627	47.30327
45.34314	50.54934	44.4771	49.56616	43.52002	48.52119	42.53689	41.51701	47.39367
45.36884	50.69975	44.51478	49.70031	43.5742	48.63828	42.60735	41.6084	47.50012
45.40992	50.86695	44.56406	49.84452	43.63891	48.76671	42.69466	41.71323	47.61615
45.46402	51.04958	44.62923	50.00333	43.71744	48.90897	42.78818	41.82977	47.74547
45.52907	51.24241	44.70602	50.17787	43.81368	49.06487	42.898	41.95996	47.88798
45.60309	51.44568	44.79639	50.35888	43.91809	49.23091	43.0183	42.09652	48.0409

EH5,1	HE8,1	EH4,1	EH3,1	HE7,1	EH2,1	EH1,1	TM0,1	HE6,1
40.15458	45.95178	39.04685	38.0829	44.72546	37.30626	36.89947	54.9386	43.47199
40.1975	45.97499	39.12841	38.19267	44.75344	37.46271	37.08395	55.22927	43.51247
40.25639	46.01661	39.22522	38.32754	44.79618	37.62828	37.27045	55.51952	43.56929
40.33312	46.06927	39.33686	38.46865	44.85793	37.79892	37.46314	55.81767	43.63875
40.42508	46.14294	39.45786	38.62249	44.9297	37.97819	37.65882	56.12319	43.72328
40.52984	46.22842	39.59214	38.78065	45.01726	38.16087	37.861	56.43158	43.81602
40.64688	46.32653	39.73773	38.94709	45.11413	38.34972	38.06334	56.74421	43.91955
40.77257	46.42407	39.91081	39.12338	45.22338	38.54559	38.27374	57.05752	44.03194
40.91139	46.55844	40.04907	39.30394	45.34263	38.74189	38.48368	57.37829	44.15122
41.06021	46.68945	40.21702	39.48882	45.47043	38.9455	38.7018	57.70861	44.28065
41.21755	46.8321	40.39259	39.68333	45.60797	39.15211	38.92134	58.03392	44.41823

HE5,1	HE4,1	HE3,1	HE2,1	HE1,1	TE0,1
42.21	41.0171	39.94842	39.11563	38.67703	57.5821
42.27158	41.11019	40.07766	39.27758	38.85862	57.8697
42.35076	41.21727	40.21099	39.44001	39.03898	58.15373
42.43987	41.32704	40.352	39.60512	39.21943	58.43583
42.53753	41.44779	40.4944	39.76916	39.39865	58.71218
42.64313	41.57436	40.64035	39.9341	39.57573	58.98448
42.75942	41.70477	40.7906	40.09837	39.75295	59.26226
42.88681	41.84034	40.94222	40.26338	39.9304	59.5318
43.01083	41.98302	41.09199	40.43065	40.1016	59.79131
43.1488	42.12445	41.24907	40.59495	40.26864	60.05121
43.28563	42.27073	41.40275	40.75919	40.43355	60.3116
