# Supplementary material for: A Photonic crystal fiber with large effective refractive index separation and low dispersion
Source: PLoS One. 2020 May 14;15(5):e0232982. doi: 10.1371/journal.pone.0232982 (PMC7224559; doi:10.1371/journal.pone.0232982)
Supplement: S2 Table — (ZIP) [file pone.0232982.s002.zip › S2 Table/changing long axis/The comparision of effective refractive index’s imaginary part.pdf]

	2	1.75	1.5	1.25	1
1.15	1.61E-17	1.96E-17	1.18E-17	7.99E-18	4.10E-18
1.2	3.15E-17	8.73E-17	2.62E-17	8.89E-18	2.74E-17
1.25	5.47E-17	4.83E-18	1.93E-17	1.97E-17	2.52E-17
1.3	4.94E-17	1.60E-17	4.25E-17	2.71E-17	4.44E-17
1.35	3.01E-17	2.91E-17	3.50E-17	2.97E-17	4.26E-17
1.4	8.55E-17	1.34E-16	1.91E-17	1.94E-17	2.66E-17
1.45	6.21E-17	1.38E-17	1.38E-17	2.59E-17	5.78E-17
1.5	1.53E-17	6.00E-17	7.50E-18	2.11E-17	4.69E-17
1.55	7.55E-17	1.30E-16	9.72E-17	2.47E-17	4.22E-17
1.6	3.62E-17	4.37E-17	8.72E-18	8.88E-18	6.37E-17
1.65	3.89E-17	1.88E-17	1.87E-17	5.72E-17	4.88E-17
