# Supplementary material for: A Photonic crystal fiber with large effective refractive index separation and low dispersion
Source: PLoS One. 2020 May 14;15(5):e0232982. doi: 10.1371/journal.pone.0232982 (PMC7224559; doi:10.1371/journal.pone.0232982)
Supplement: S2 Table — (ZIP) [file pone.0232982.s002.zip › S2 Table/changing long axis/The comparision of effective refractive index’s real part.pdf]

	2	1.75	1.5	1.25	1
1.15	1.7727	1.7728	1.7728	1.7728	1.7728
1.2	1.7706	1.7707	1.7707	1.7707	1.7707
1.25	1.7685	1.7686	1.7686	1.7686	1.7686
1.3	1.7663	1.7664	1.7664	1.7664	1.7664
1.35	1.764	1.7641	1.7641	1.7642	1.7642
1.4	1.7618	1.7618	1.7619	1.7619	1.7619
1.45	1.7594	1.7595	1.7595	1.7595	1.7596
1.5	1.757	1.7571	1.7571	1.7572	1.7572
1.55	1.7546	1.7547	1.7547	1.7548	1.7548
1.6	1.7521	1.7523	1.7523	1.7523	1.7524
1.65	1.7496	1.7498	1.7498	1.7498	1.7499
