# Supplementary material for: A Photonic crystal fiber with large effective refractive index separation and low dispersion
Source: PLoS One. 2020 May 14;15(5):e0232982. doi: 10.1371/journal.pone.0232982 (PMC7224559; doi:10.1371/journal.pone.0232982)
Supplement: S2 Table — (ZIP) [file pone.0232982.s002.zip › S2 Table/changing long axis/The comparision of HE41’s despersion.pdf]

	2	1.75	1.5	1.25	1
1.15	-67.764	-53.827	-72.233	-91.533	-63.303
1.2	-41.804	-31.736	-46.654	-62.691	-39.2
1.25	-20.541	-14.715	-25.787	-38.218	-19.962
1.3	-3.138	-1.926	-8.795	-17.279	-4.757
1.35	11.048	7.271	4.963	0.768	7.06
1.4	22.516	13.379	15.989	16.424	15.989
1.45	31.662	16.791	24.677	30.085	22.425
1.5	38.8	17.822	31.341	42.063	26.682
1.55	44.18	16.723	36.233	52.611	29.011
1.6	48.008	13.698	39.556	61.933	29.616
1.65	50.45	8.914	41.478	70.194	28.665
