# Supplementary material for: A Photonic crystal fiber with large effective refractive index separation and low dispersion
Source: PLoS One. 2020 May 14;15(5):e0232982. doi: 10.1371/journal.pone.0232982 (PMC7224559; doi:10.1371/journal.pone.0232982)
Supplement: S2 Table — (ZIP) [file pone.0232982.s002.zip › S2 Table/changing long axis/The comparision of TE01’s confinement loss.pdf]

	2	1.75	1.5	1.25	1
1.15	7.66E-10	9.31E-10	5.58E-10	3.79E-10	1.95E-10
1.2	1.43E-09	3.97E-09	1.19E-09	4.04E-10	1.24E-09
1.25	2.39E-09	2.11E-10	8.43E-10	8.58E-10	1.1E-09
1.3	2.07E-09	6.7E-10	1.78E-09	1.14E-09	1.86E-09
1.35	1.22E-09	1.18E-09	1.41E-09	1.2E-09	1.72E-09
1.4	3.33E-09	5.21E-09	7.44E-10	7.57E-10	1.04E-09
1.45	2.34E-09	5.21E-10	5.21E-10	9.75E-10	2.17E-09
1.5	5.56E-10	2.18E-09	2.73E-10	7.69E-10	1.71E-09
1.55	2.66E-09	4.57E-09	3.42E-09	8.71E-10	1.49E-09
1.6	1.23E-09	1.49E-09	2.98E-10	3.03E-10	2.17E-09
1.65	1.29E-09	6.2E-10	6.2E-10	1.89E-09	1.61E-09
