# Supplementary material for: A Photonic crystal fiber with large effective refractive index separation and low dispersion
Source: PLoS One. 2020 May 14;15(5):e0232982. doi: 10.1371/journal.pone.0232982 (PMC7224559; doi:10.1371/journal.pone.0232982)
Supplement: S2 Table — (ZIP) [file pone.0232982.s002.zip › S2 Table/changing long axis/The comparision of TE01’s despersion.pdf]

	2	1.75	1.5	1.25	1
1.15	-91.625	-81.167	-77.601	-99.582	-95
1.2	-64.632	-58.214	-54.492	-71.648	-68.718
1.25	-42.163	-40.16	-36.281	-48.129	-47.006
1.3	-23.383	-26.168	-22.131	-28.192	-29.029
1.35	-7.647	-15.595	-11.402	-11.192	-14.145
1.4	5.544	-7.942	-3.591	3.371	-1.852
1.45	16.585	-2.813	1.695	15.891	8.244
1.5	25.791	0.105	4.771	26.682	16.458
1.55	33.412	1.064	5.889	35.997	23.04
1.6	39.652	0.269	5.251	44.039	28.195
1.65	44.679	-2.115	3.026	50.974	32.089
