# Supplementary material for: A Photonic crystal fiber with large effective refractive index separation and low dispersion
Source: PLoS One. 2020 May 14;15(5):e0232982. doi: 10.1371/journal.pone.0232982 (PMC7224559; doi:10.1371/journal.pone.0232982)
Supplement: S2 Table — (ZIP) [file pone.0232982.s002.zip › S2 Table/changing long axis/The comparison of effective refractive index separation between EH1-HE3 modes.pdf]

	2	1.75	1.5	1.25	1
1.15	0.0046	0.0046	0.0046	0.0046	0.0046
1.2	0.0053	0.0052	0.0051	0.0052	0.0052
1.25	0.0059	0.0059	0.0059	0.0058	0.0058
1.3	0.0066	0.0065	0.0065	0.0066	0.0065
1.35	0.0074	0.0073	0.0073	0.0073	0.0072
1.4	0.0081	0.008	0.008	0.008	0.008
1.45	0.0089	0.0089	0.0089	0.0089	0.0088
1.5	0.0099	0.0098	0.0098	0.0097	0.0097
1.55	0.0108	0.0107	0.0107	0.0106	0.0106
1.6	0.0118	0.0117	0.0117	0.0116	0.0116
1.65	0.0128	0.0127	0.0127	0.0127	0.0127
