# Supplementary material for: A Photonic crystal fiber with large effective refractive index separation and low dispersion
Source: PLoS One. 2020 May 14;15(5):e0232982. doi: 10.1371/journal.pone.0232982 (PMC7224559; doi:10.1371/journal.pone.0232982)
Supplement: S2 Table — (ZIP) [file pone.0232982.s002.zip › S2 Table/changing short axis/The comparision of effective refractive index’s imaginarypart in TE01 mode.pdf]

	4	3.5-7	3*7	2.5-7	2*7
1.15	1.61E-17	1.62E-17	3.40E-17	1.40E-17	3.21E-18
1.2	3.15E-17	4.51E-17	2.10E-17	3.89E-18	1.78E-17
1.25	5.47E-17	2.49E-17	1.86E-17	4.30E-18	1.18E-17
1.3	4.94E-17	6.04E-17	5.12E-18	4.73E-18	4.34E-17
1.35	3.01E-17	6.02E-18	2.24E-17	3.11E-17	9.49E-18
1.4	8.55E-17	4.60E-17	6.12E-18	2.26E-17	6.21E-17
1.45	6.21E-17	1.55E-17	2.00E-17	1.23E-17	5.62E-18
1.5	1.53E-17	5.27E-18	2.16E-17	6.65E-18	6.09E-17
1.55	7.55E-17	4.39E-17	2.34E-17	3.59E-17	7.88E-17
1.6	3.62E-17	9.00E-18	5.03E-17	7.73E-17	4.95E-17
1.65	3.89E-17	8.91E-18	1.80E-17	9.13E-17	7.58E-17
