# Supplementary material for: A Photonic crystal fiber with large effective refractive index separation and low dispersion
Source: PLoS One. 2020 May 14;15(5):e0232982. doi: 10.1371/journal.pone.0232982 (PMC7224559; doi:10.1371/journal.pone.0232982)
Supplement: S2 Table — (ZIP) [file pone.0232982.s002.zip › S2 Table/changing short axis/The comparision of effective refractive index’s real part in HE41 mode.pdf]

	4比7	3.5比7	3比7	2.5比7	2比7
1.15	1.7704	1.7705	1.7707	1.7708	1.7709
1.2	1.7681	1.7682	1.7684	1.7685	1.7687
1.25	1.7657	1.7659	1.766	1.7662	1.7663
1.3	1.7633	1.7634	1.7636	1.7638	1.764
1.35	1.7608	1.761	1.7611	1.7613	1.7615
1.4	1.7582	1.7584	1.7586	1.7588	1.7591
1.45	1.7556	1.7558	1.756	1.7563	1.7565
1.5	1.7529	1.7532	1.7534	1.7537	1.7539
1.55	1.7502	1.7505	1.7507	1.751	1.7513
1.6	1.7475	1.7477	1.748	1.7483	1.7486
1.65	1.7447	1.745	1.7452	1.7456	1.7459
