# Supplementary material for: A Photonic crystal fiber with large effective refractive index separation and low dispersion
Source: PLoS One. 2020 May 14;15(5):e0232982. doi: 10.1371/journal.pone.0232982 (PMC7224559; doi:10.1371/journal.pone.0232982)
Supplement: S2 Table — (ZIP) [file pone.0232982.s002.zip › S2 Table/changing short axis/The comparision of HE41’s dispersion.pdf]

	4比7	3.5比7	3比7	2.5比7	2比7
1.15	-53.827	-63.834	-107.066	-89.748	-79.382
1.2	-31.736	-39.568	-76.852	-62.319	-53.368
1.25	-14.715	-20.153	-50.836	-39.385	-32.003
1.3	-1.926	-4.755	-28.184	-20.109	-14.452
1.35	7.271	7.271	-8.253	-3.848	-0.072
1.4	13.379	16.424	9.459	9.897	11.638
1.45	16.791	23.1	25.344	21.523	21.072
1.5	17.822	27.612	39.719	31.341	28.544
1.55	16.723	30.211	52.833	39.606	34.306
1.6	13.698	31.103	64.892	46.519	38.562
1.65	8.914	30.453	76.061	52.248	41.479
