# Supplementary material for: A Photonic crystal fiber with large effective refractive index separation and low dispersion
Source: PLoS One. 2020 May 14;15(5):e0232982. doi: 10.1371/journal.pone.0232982 (PMC7224559; doi:10.1371/journal.pone.0232982)
Supplement: S2 Table — (ZIP) [file pone.0232982.s002.zip › S2 Table/changing short axis/The comparision of TE01’s confinement loss.pdf]

	4*7	3.5-7	3*7	2.5-7	2*7
1.15	7.66E-10	7.7E-10	1.62E-09	6.65E-10	1.52E-10
1.2	1.43E-09	2.05E-09	9.56E-10	1.77E-10	8.11E-10
1.25	2.39E-09	1.09E-09	8.12E-10	1.88E-10	5.16E-10
1.3	2.07E-09	2.54E-09	2.15E-10	1.99E-10	1.82E-09
1.35	1.22E-09	2.43E-10	9.07E-10	1.26E-09	3.84E-10
1.4	3.33E-09	1.79E-09	2.39E-10	8.81E-10	2.42E-09
1.45	2.34E-09	5.83E-10	7.51E-10	4.62E-10	2.12E-10
1.5	5.56E-10	1.92E-10	7.87E-10	2.42E-10	2.21E-09
1.55	2.66E-09	1.55E-09	8.22E-10	1.26E-09	2.78E-09
1.6	1.23E-09	3.07E-10	1.71E-09	2.64E-09	1.69E-09
1.65	1.29E-09	2.95E-10	5.95E-10	3.02E-09	2.51E-09
