# Supplementary material for: A Photonic crystal fiber with large effective refractive index separation and low dispersion
Source: PLoS One. 2020 May 14;15(5):e0232982. doi: 10.1371/journal.pone.0232982 (PMC7224559; doi:10.1371/journal.pone.0232982)
Supplement: S2 Table — (ZIP) [file pone.0232982.s002.zip › S2 Table/changing short axis/The comparision of TE01’s dispersion.pdf]

	4比7	3.5比7	3比7	2.5比7	2比7
1.15	-81.167	-132.638	-106.543	-88.318	-123.161
1.2	-58.214	-99.615	-78.353	-63.811	-94.016
1.25	-40.16	-70.464	-54.534	-44.046	-69.101
1.3	-26.168	-44.351	-34.248	-28.189	-47.581
1.35	-15.595	-20.631	-16.854	-15.596	-28.812
1.4	-7.942	1.195	-1.851	-5.767	-12.294
1.45	-2.813	21.523	11.157	1.694	2.368
1.5	0.105	40.665	22.482	7.099	15.488
1.55	1.064	58.875	32.376	10.701	27.317
1.6	0.269	76.355	41.045	12.704	38.059
1.65	-2.115	93.273	48.653	13.274	47.882
